# Impact of dipeptidyl peptidase-4 inhibitors on glucose-dependent insulinotropic polypeptide in type 2 diabetes mellitus: a systematic review and meta-analysis

**DOI:** 10.3389/fendo.2023.1203187

**Published:** 2023-08-10

**Authors:** Shangyu Chai, Ruya Zhang, Richard David Carr, Carolyn F. Deacon, Yiman Zheng, Swapnil Rajpathak, Jingya Chen, Miao Yu

**Affiliations:** ^1^ Merck Research Laboratories (MRL) Global Medical Affairs, Merck Sharp & Dohme (MSD) China, Shanghai, China; ^2^ Hatter Cardiovascular Institute, University College London, London, United Kingdom; ^3^ School of Biomedical Sciences, Ulster University, Coleraine, United Kingdom; ^4^ Department of Biomedical Sciences, University of Copenhagen, Copenhagen, Denmark; ^5^ Merck Research Laboratories, Merck & Co., Inc., Rahway, NJ, United States; ^6^ Department of Endocrinology, Key Laboratory of Endocrinology, National Health Commission, Peking Union Medical College Hospital, Peking Union Medical College and Chinese Academy of Medical Sciences, Beijing, China

**Keywords:** dipeptidyl peptidase-4 inhibitor, glucose-dependent insulinotropic polypeptide, metabolism, randomized controlled trials, meta-analysis

## Abstract

**Aims:**

Glucose-dependent insulinotropic polypeptide (GIP) confers a variety of metabolic benefits in type 2 diabetes mellitus (T2DM). This meta-analysis was conducted to investigate the impact of dipeptidyl peptidase 4 (DPP4) inhibitors on GIP levels in T2DM patients.

**Methods:**

Medline (PubMed), CENTER (Cochrane Library), and Embase (Ovid) were searched and randomized controlled trials (RCTs) evaluating the impact of DPP4 inhibitors on fasting and postprandial GIP levels were obtained. For postprandial GIP, only studies with the data of GIP changes reported as the total area under the curve (AUC_GIP_) using a meal or oral glucose tolerance test were included. A random-effects model was used for data pooling after incorporating heterogeneity.

**Results:**

Overall, 14 RCTs with 541 T2DM patients were included. Compared to placebo/no treatment, the use of DPP4 inhibitors significantly increased the fasting GIP level (standard mean difference [SMD]: 0.77, 95% confidence interval [CI]: 0.48–1.05, *P*<0.001; *I^2 = ^* 52%) and postprandial AUC_GIP_ (SMD: 1.33, 95% CI: 1.02–1.64, *P*<0.001; *I^2 = ^* 65%). Influence analysis by excluding one dataset at a time showed consistent results. Sensitivity analyses only including studies with radioimmunoassay showed also consistent results (fasting GIP: SMD: 0.75, 95% CI: 0.51–1.00, P<0.001; I^2 = ^0%; and postprandial AUC_GIP_: SMD: 1.48, 95% CI: 1.18–1.78, *P*<0.001; *I^2 = ^* 54%). Further subgroup analyses demonstrated that the influence of DPP4 inhibitors on fasting and postprandial GIP levels in T2DM patients was not significantly changed by study characteristics such as study design, patient mean age, baseline glycated hemoglobin (HbA1c) concentration, body mass index (BMI), background treatment, treatment duration, or method for postprandial GIP measurement (all *P* for subgroup effects <0.05).

**Conclusion:**

The use of DPP4 inhibitors effectively increases the fasting and postprandial GIP concentrations in T2DM patients.

**Systematic review registration:**

https://www.crd.york.ac.uk/prospero/, identifier CRD42022356716.

## Introduction

Increasing evidence suggests that incretin hormones, such as glucose-dependent insulinotropic polypeptide (GIP) and glucagon-like peptide-1 (GLP-1), are actively involved in glucose regulation (1). GIP and GLP-1 are secreted by intestinal enteroendocrine cells, which can improve glycemic control by enhancing insulin secretion, preventing glucagon release, augmenting glucose sensitivity, attenuating hepatic glucose production, and stimulating peripheral glucose utilization in adipose tissue and muscles (2, 3). In addition, GIP and GLP-1 have been shown to optimize lipid metabolism and endothelial function, regulate appetite and satiety, and increase myocardial contractility (4). Although the drug development and marketing of GIP as a therapeutic agent lags far behind those for GLP-1, increasing evidence has showed the ability of GIP to improve glucose and lipid metabolism (5), particularly when paired with the mechanism of GLP-1. However, although GIP is among the predominant incretin hormones in healthy population (3), the insulin response to GIP in subjects with type 2 diabetes mellitus (T2DM) is lower than that for GLP-1 (4). Unlike GLP-1, GIP does not affect glucagon concentrations during hyperglycemia, but it has been confirmed that it increases glucagon levels under both fasting and hypoglycemic conditions, which may contribute to reducing the risk of severe hypoglycemia in T2DM patients (5). In this regard, harnessing the benefits of GIP in patients with T2DM may be advantageous when seeking to improve glycemic control and treat metabolic disorders.

Dipeptidyl peptidase 4 (DPP4) inhibitors are commonly prescribed antidiabetic drugs (OADs) that may provide additional benefits besides glucose-lowering effects, such as attenuating β-cell loss, inhibiting glucagon secretion, reducing glucose fluctuation, and improving glycemic durability during the progression of T2DM (6–8). Moreover, DPP4 inhibitors have overall good safety profile and tolerability (7). The pharmacological mechanism of DPP4 inhibitors relies on the restoration of incretin hormone levels in T2DM patients (7), and it is well established that they increase GLP-1 levels (9). However, influence of DPP4 inhibitors on GIP levels in T2DM patients has not gained similar attention (10), perhaps due to the known impaired insulinotropic effect of GIP in these subjects (4). On the other hand, some small-scale randomized controlled trials (RCTs) showed inconsistent results as for the influence of DPP4 inhibitors on GIP levels (11–24). Therefore, we performed a systematic review and meta-analysis to comprehensively evaluate the efficacy of DPP4 inhibitors on fasting and postprandial GIP in patients with T2DM.

## Methods

This study is in accordance with the guidelines of Preferred Reporting Items for Systematic Reviews and Meta-Analyses (25, 26) and Cochrane Handbook (27). The protocol was prospectively registered at PROSPERO (https://www.crd.york.ac.uk/prospero/) with the code CRD42022356716.

### Search strategy

A combined search strategy was used for study identification in Medline (PubMed), CENTER (Cochrane Library), and Embase (Ovid), which included: (1) “sitagliptin” OR “vildagliptin” OR “linagliptin” OR “trelagliptin” OR “omarigliptin” OR “anagliptin” OR “teneligliptin” OR “saxagliptin” OR “alogliptin” OR “gemigliptin” OR “evogliptin” OR “dutogliptin” OR “aemigliptin” OR “DPP-4” OR “DPP4” OR “dipeptidyl peptidase-4 inhibitors”; (2) “GIP” OR “glucose-dependent insulinotropic polypeptide” OR “gastric inhibitory peptide” OR “incretin” OR “hormone” OR “hormonal” OR “postprandial” OR “oral glucose tolerance test” OR “meal” OR “prandial” OR “OGTT;” and (3) “randomly” OR “placebo” OR “allocated” OR “control” OR “randomized” OR “randomised” OR “random”. We only considered studies including human subjects. We also manually searched the references to related reviews and original articles. The date of the last database search was June 16, 2022.

### Study selection

The PICOS principle, described below, was followed in designating the inclusion criteria of the meta-analysis. P (patients): Adult patients with T2DM; I (intervention): Oral DPP4 inhibitors with approved dosages; C (control): Placebo or no treatment; O (outcomes): Between-group difference of changes of either the fasting intact/active GIP level or postprandial GIP level from baseline as the total area under the curve (AUC_GIP_) using the meal tolerance test or the oral glucose tolerance test. Specifically, GIP is secreted as an intact 42-amino acid peptide, which is rapidly degraded by dipeptidyl peptidase 4 into inactive GIP (3–42) (28). To keep consistency, we only included studies reporting the serum concentrations of intact/active GIP. Only studies of full-length articles in English were considered eligible. Studies with single-dose/single-day DPP4 inhibitor treatment were excluded because we did not want to observe the acute effects of DPP4 inhibitors on GIP levels. Additionally, studies with T2DM patients receiving oral GLP-1 receptor agonists (GLP-1RAs) or concurrent injectable antidiabetic treatment, such as injectable GLP-1RAs or insulin, were excluded from the current meta-analysis. Moreover, nonrandomized studies, studies including non-T2DM patients, studies comparing DPP4 inhibitors with active controls, and studies that did not report GIP concentrations were also excluded.

### Data extraction and quality evaluation

Two authors independently searched databases, collected data, and evaluated quality. Whenever disagreements arose, the corresponding author was consulted. The following data were collected: study general information, study design characteristics, patient characteristics (sample size, age, gender, baseline glycated hemoglobin [HbA1c], body mass index [BMI], and T2DM duration), previous antidiabetic treatments, drug name and dose of the DPP4 inhibitor used, regimen of the controls, treatment duration, and method for measuring circulating GIP. Quality of RCTs included in this review was assessed using the Cochrane Risk of Bias Tool (27) involving seven domains: production of random sequence, concealing of allocations, blinding to the participants and personnel, blinding of outcomes evaluation, incomplete result data, and selective reported outcomes.

### Statistical analysis

The impacts of DPP4 inhibitors on fasting GIP and postprandial AUC_GIP_ in T2DM patients were calculated as the standard mean difference (SMD) with the 95% confidence interval (CI), because of the inconsistent methods for GIP measuring. Heterogeneity was investigated using Cochrane Q test (27). In addition, the *I^2^* statistic was determined, with *I^2^* >50% indicating significant heterogeneity (29). A random-effects model was used for data pooling by incorporating possible heterogeneity (27). Influence analysis was performed by “leaving one study out” from the meta-analysis at a time (27). Additionally, since the radioimmunoassay (antibody 98171) was the most well-characterized for intact GIP (30) and has been most broadly applied in previous studies, sensitivity analysis limited to studies using this immunoassay was performed (27). Analysis of predefined subgroups was also performed according to predefined study features. Egger’s regression asymmetry test and funnel plots were used to assess publication bias (31). In studies with multiple DPP4 inhibitor interventions or dose groups, the control groups were split equally. The purpose was to overcome unit-of-analysis errors as detailed in Cochrane Handbook (27). *P*<0.05 indicated a statistically significant difference. Statistics were carried out with RevMan software (Version 5.1; Cochrane, Oxford, UK).

## Results

### Literature search

**Figure 1:**
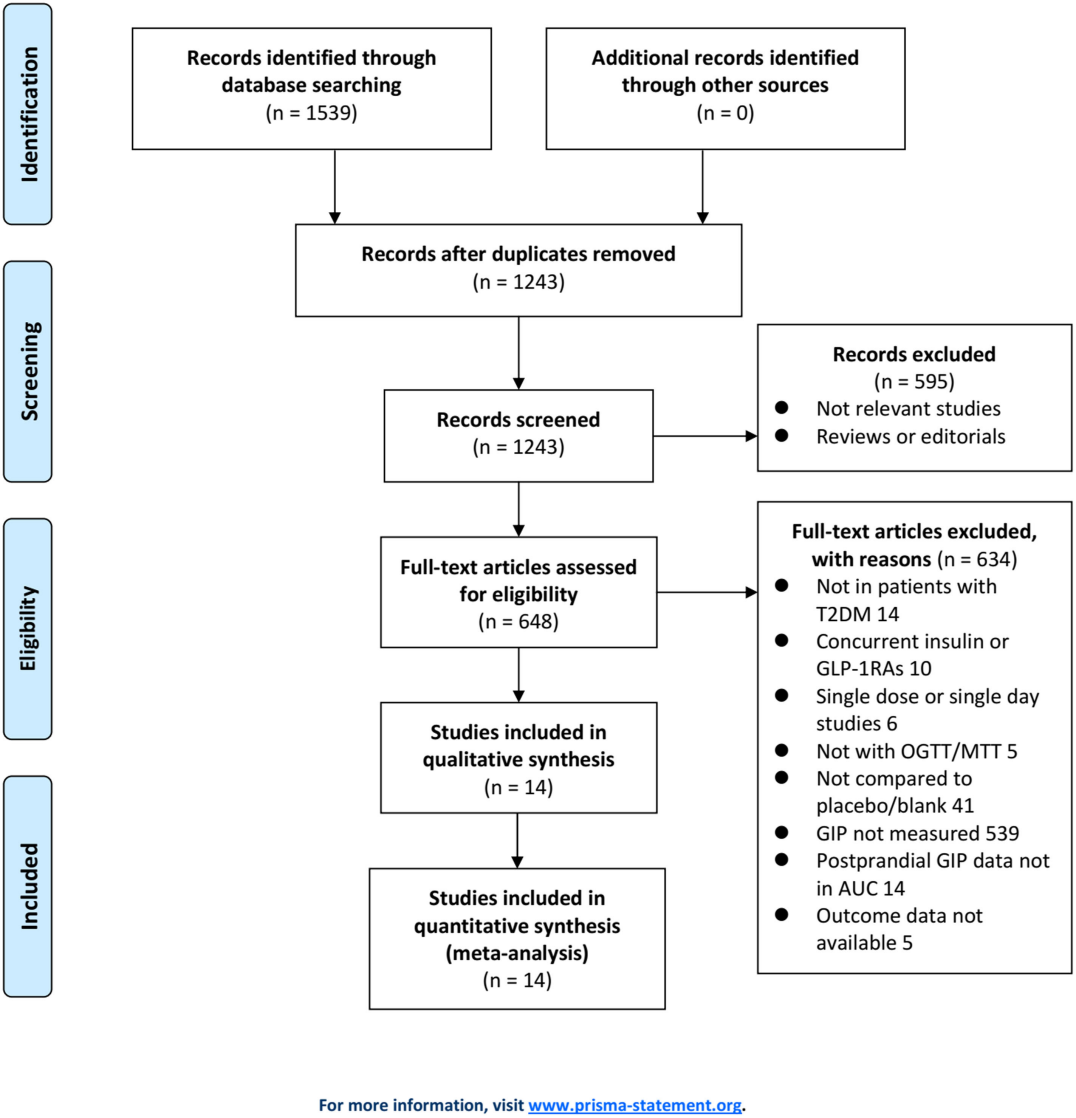
Flowchart of the literature search.

**Figure 1** displays the procedure of literature obtaining. In brief, database searches retrieved 1539 articles, and 1243 were yielded after excluding duplications. A total of 595 articles were then removed based on the titles and abstracts, for they were unrelated to the study aim. Subsequently, 634 out of the 648 articles were further excluded after full text reading for the reasons presented in **Figure 1**. Finally, 14 RCTs (11–24) were used for the meta-analysis.

### Study characteristics and data quality

An overview of the included studies is shown in **Table 1**. Overall, 14 RCTs including 541 patients with T2DM were available for the meta-analysis. These studies were published between 2007 and 2018, and performed in the United States (11–14, 19), Canada (15), Germany (16, 18, 21, 22), Italy (17), Sweden (24), Japan (20), and Korea (23). Nine of them were crossover studies (11–13, 15, 16, 19, 21, 22, 24), while the remaining five studies (14, 17, 18, 20, 23) were parallel-group RCTs. The mean ages of the patients varied between 47 and 74 years old. The baseline HbA1c varied between 6.6% and 8.8%, and the baseline BMI ranged from 22.5 to 33.9 kg/m^2^. Various DPP4 inhibitors were used among these studies, such as sitagliptin, vildagliptin, linagliptin, and evogliptin, while placebo was used as the control in all of the included RCTs except for one study that had one group receiving no treatment (20). The treatment durations varied between 6 and 168 days. The outcomes of fasting GIP were reported in eight studies (11–15, 19, 23, 24), while the outcomes of postprandial AUC_GIP_ were reported in 12 studies (11–13, 15–18, 20–24). A radioimmunoassay (11–19, 21, 22) or an enzyme-linked immunosorbent assay (24) was used for measuring intact GIP in 12 studies, while active GIP was measured in the other two studies (20, 23). The units of GIP concentration in each study are shown in **Table 1**. **Table 2** provides a detailed analysis of the included RCTs using Cochrane’s Risk of Bias Tool. All of the included studies were double-blind except for one study, which was open label (20). The details of the random sequence generation were only reported in one study (19), while none of the included RCTs reported the details of allocation concealment.

**Table 1:** Characteristics of the included studies.

Study	Country	Design	Patient number	Mean age	Male	HbA1c	BMI	T2DM duration	DPP4i and dose	Controls	Drug naive	Duration	Timing of GIP measurements and units	Method for GIP measurements
				years	%	%	kg/m^2^	years				days		
He 2007	USA	R, DB, PC, CO	13	53.5	46.2	7.5–10	NR	7.2	Vildagliptin 10, 25, or 100 mg Bid	Placebo	Partial	28	Fasting (pmol/L) and MTT-AUC (4 h, pmol h/L)	RIA, Antiserum 98171 for intact GIP
Vella 2008	USA	R, DB, PC, CO	14	53.1	NR	8	33.9	NR	Vildagliptin 50 mg Bid	Placebo	NR	10	Fasting (pmol/L) and MTT-AUC (3 h, nmol 3h/L)	RIA, Antiserum 98171 for intact GIP
Azuma 2008	USA	R, DB, PC, CO	16	56	56.3	7.1	31.4	NR	Vildagliptin 50 mg Bid	Placebo	Partial	42	Fasting (pmol/L) and MTT-AUC (5 h, pmol 5h/L)	RIA, Antiserum 98171 for intact GIP
D’Alessio 2009	USA	R, DB, PC	39	55	59	6.6	32.3	3.6	Vildagliptin 50 mg Bid	Placebo	None	84	Fasting (pmol/L)	RIA, Antiserum 98171 for intact GIP
Tremblay 2011	Canada	R, DB, PC, CO	36	58.1	83.3	6.8	30.7	NR	Sitagliptin 100 mg Qd	Placebo	None	42	Fasting (pmol/L) and MTT-AUC (8 h, pmol h/L)	RIA, Antiserum 98171 for intact GIP
Vardarli 2011	Germany	R, DB, PC, CO	21	59	85.7	7.3	28.6	6	Vildagliptin 100 mg Qd	Placebo	None	13	OGTT-AUC (4 h, pmol 4h/L)	RIA, Antiserum 98171 for intact GIP
Rauch 2012	Germany	R, DB, PC	80	NR	NR	7.3	NR	NR	Linagliptin 5 mg Qd	Placebo	Partial	28	MTT-AUC (2 h, pmol h/L)	RIA, Antiserum 98171 for intact GIP
Muscelli 2012	Italy	R, DB, PC	47	56.1	29.8	7.4	29.9	NR	Sitagliptin 100 mg Qd	Placebo	Partial	42	MTT-AUC (5 h, nmol 5h/L)	RIA, Antiserum 98171 for intact GIP
Solis 2013	USA	R, DB, PC, CO	16	47	56.3	8.8	33.5	1.5	Sitagliptin 100 mg Qd	Placebo	Partial	42	Fasting (pg/mL)	RIA, Antiserum 98171 for intact GIP
Vardarli 2014	Germany	R, DB, PC, CO	20	59	80	7.0	30.6	5	Sitagliptin 100 mg Qd	Placebo	Partial	6	OGTT-AUC (4 h, pmol 4h/L)	RIA, Antiserum 98171 for intact GIP
Mikada 2014	Japan	R, OL	27	59.6	66.7	7.0	28.9	8.4	Sitagliptin 50 mg Qd	No treatment	Partial	168	MTT-AUC (2 h, pmol h/L)	LC-MS/MS for active GIP
Baranov 2016	Germany	R, DB, PC, CO	24	63	58.3	6.6	30	5.4	Vildagliptin 50 mg Bid or sitagliptin 100 mg Qd	Placebo	Partial	9	MTT-AUC (4 h, pmol 4h/L))	RIA, Antiserum 98171 for intact GIP
Park 2017	Korea	R, DB, PC	160	57.2	53.1	7.2	25.5	4.5	Evogliptin 5 mg Qd	Placebo	Partial	168	Fasting (pg/mL) and OGTT-AUC (2 h, pg 2h/mL)	NR, active GIP
Farngren 2018	Sweden	R, DB, PC, CO	28	74	60.7	6.9	30.2	9.2	Sitagliptin 100 mg Qd	Placebo	None	28	Fasting (nmol/L) and MTT-AUC (2 h, nmol/L min)	ELISA, intact GIP

BMI, body mass index; T2DM, type 2 diabetes mellitus; DPP4i, dipeptidyl peptidase-4 inhibitors; GIP, glucose-dependent insulinotropic polypeptide; R, randomized; DB, double-blind; PC, placebo-controlled; CO, crossover; NR, not reported; Bid, twice daily; Qd, once daily; MTT, meal tolerance test; OGTT, oral glucose tolerance test; AUC, area under the curve; RIA, radioimmunoassay; LC-MS/MS, liquid chromatography-tandem mass spectrometry; ELISA, enzyme-linked immunosorbent assay; HbA1c, glycosylated hemoglobin; BMI, body mass index.

**Table 2:** Study quality evaluation via the cochrane’s risk of bias tool.

Study	Random sequence generation	Allocation concealment	Blinding of participants	Blinding of outcome assessment	Incomplete outcome data addressed	Selective reporting	Other sources of bias
He 2007	Unclear	Unclear	Low	Low	Low	Low	Low
Vella 2008	Unclear	Unclear	Low	Low	Low	Low	Low
Azuma 2008	Unclear	Unclear	Low	Low	Low	Low	Low
D’Alessio 2009	Unclear	Unclear	Low	Low	Low	Low	Low
Tremblay 2011	Unclear	Unclear	Low	Low	Low	Low	Low
Vardarli 2011	Unclear	Unclear	Low	Low	Low	Low	Low
Rauch 2012	Unclear	Unclear	Low	Low	Low	Low	Low
Muscelli 2012	Unclear	Unclear	Low	Low	Low	Low	Low
Solis 2013	Low	Unclear	Low	Low	Low	Low	Low
Vardarli 2014	Unclear	Unclear	Low	Low	Low	Low	Low
Mikada 2014	Unclear	Unclear	High	High	Low	Low	Low
Baranov 2016	Unclear	Unclear	Low	Low	Low	Low	Low
Park 2017	Unclear	Unclear	Low	Low	Low	Low	Low
Farngren 2018	Unclear	Unclear	Low	Low	Low	Low	Low

### Influence of DPP4 inhibitors on fasting GIP

**Figure 2:**
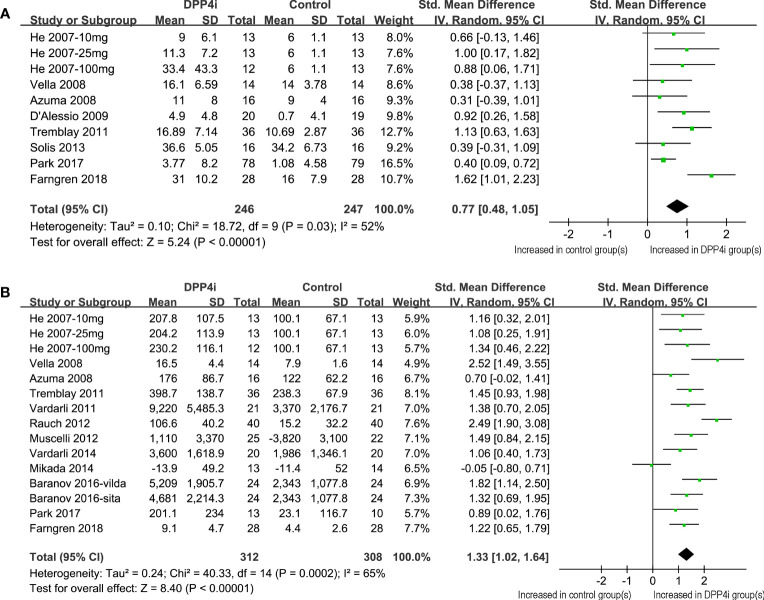
Forest plots for the meta-analysis comparing the effects of DPP4 inhibitors with controls on the circulating GIP levels in T2DM patients. **(A)** Forest plots for the influence of DPP4 inhibitors on fasting GIP; and **(B)** forest plots for the influence of DPP4 inhibitors on postprandial AUC_GIP_.

Because one study reported data following three dosage levels of vildagliptin separately, these datasets were included in the meta-analysis independently (11). Accordingly, 10 datasets from eight RCTs (11–15, 19, 23, 24) were available for the evaluation of the influence of DPP4 inhibitors on fasting GIP levels in T2DM patients. Overall, the results of the meta-analysis showed that compared to placebo, the use of DPP4 inhibitors significantly increased the fasting GIP level in these patients (SMD: 0.77, 95% CI: 0.48–1.05, *P*<0.001; *I^2 = ^* 52%; **Figure 2A**). Influence analysis showed consistent results (SMD: 0.64–0.84, all *P*<0.05) after excluding one dataset at a time. Sensitivity analyses also demonstrated similar results limited to studies using a radioimmunoassay (SMD: 0.75, 95% CI: 0.51–1.00, *P*<0.001; *I^2 = ^* 0%). In addition, DPP-4 inhibitors significantly increased fasting GIP concentrations in all subgroup analyses by study characteristics such as study design, mean age, baseline HbA1c, BMI, background treatment, or treatment duration (**Table 3**, all *P* for subgroup effects <0.05). However, DPP-4 inhibitors increased fasting GIP to a greater extent in patients with background OADs as compared to patients who were drug-naive or without background OADs (**Table 3**, *P* for subgroup difference = 0.001)

**Table 3:** Subgroup analysis for comparing DPP4 inhibitors with placebo/no treatment on fasting GIP.

	Datasets	SMD [95% CI]	*P* for subgroup effect	*I^2^*	*P* for subgroup difference
Design					
Crossover	8	0.83 [0.49, 1.16]	<0.001	47%	
Parallel group	2	0.58 [0.10, 1.05]	0.02	47%	0.40
Mean age (years)					
≤54	5	0.63 [0.29, 0.98]	<0.001	0%	
>54	5	0.86 [0.38, 1.35]	<0.001	76%	0.45
Baseline HbA1c (%)					
≤7.5	5	0.86 [0.38, 1.35]	<0.001	76%	
>7.5	5	0.63 [0.29, 0.98]	<0.001	0%	0.45
BMI (kg/m^2^)					
≤32	4	0.86 [0.27, 1.44]	0.004	81%	
>32	3	0.59 [0.18, 0.99]	0.005	0%	0.46
Background treatment					
With OADs	3	1.22 [0.84, 1.61]	<0.001	23%	
Drug naive or without OADs	6	0.50 [0.27, 0.73]	<0.001	0%	0.001
Treatment duration (days)					
≤28	5	0.94 [0.48, 1.40]	<0.001	46%	
>28	5	0.63 [0.30, 0.97]	<0.001	48%	0.28

DPP4, dipeptidyl peptidase 4; GIP, glucose dependent insulinotropic polypeptide; SMD, standard mean difference; CI, confidence interval; HbA1c, glycosylated hemoglobin; BMI, body mass index; OADs, oral antidiabetic drugs; NA, not applicable.

### Influence of DPP4 inhibitors on postprandial AUC_GIP_

Overall, 15 datasets from 12 RCTs were included for the meta-analysis evaluating the influence of DPP4 inhibitors on postprandial AUC_GIP_ in T2DM patients. The pooled results showed that compared to placebo or no treatment, the use of DPP4 inhibitors significantly increased AUC_GIP_ in these patients (SMD: 1.33, 95% CI: 1.02–1.64, *P*<0.001; *I^2 = ^* 65%; **Figure 2B**). Influence analysis by excluding one dataset at a time showed consistent results (SMD: 1.23–1.42, all *P*<0.05). Sensitivity analyses limited to studies using a radioimmunoassay (SMD: 1.48, 95% CI: 1.18–1.78, *P*<0.001; *I^2 = ^* 54%) also showed similar results. Further subgroup analyses revealed that the influence of DPP4 inhibitors on postprandial AUC_GIP_ in T2DM patients was not significantly affected by the study characteristics, including study design, mean age, baseline HbA1c, BMI, background treatment, treatment duration, or method for postprandial AUC_GIP_ measurement (**Table 4**, all *P* for subgroup effects <0.05, all P for subgroup differences >0.05).

**Table 4:** Subgroup analysis for comparing DPP4 inhibitors with placebo/no treatment on postprandial AUC_GIP_.

	Datasets	SMD (95% CI)	*P* for subgroup effect	*I^2^*	*P* for subgroup difference
Design					
Crossover	11	1.33 [1.10, 1.56]	<0.001	14%	
Parallel group	4	1.23 [0.14, 2.31]	0.03	89%	0.85
Mean age (years)					
≤58	8	1.35 [1.05, 1.65]	<0.001	23%	
>58	6	1.07 [0.60, 1.54]	<0.001	64%	0.33
Baseline HbA1c (%)					
≤7.5	4	1.47 [0.87, 2.07]	<0.001	45%	
>7.5	11	1.28 [0.91, 1.65]	<0.001	71%	0.59
BMI (kg/m^2^)					
≤30	6	1.17 [0.66, 1.67]	<0.001	67%	
>30	5	1.31 [0.86, 1.76]	<0.001	56%	0.68
Background treatment					
With OADs	3	1.35 [1.02, 1.69]	<0.001	0%	
Drug naive or without OADs	11	1.23 [0.82, 1.64]	<0.001	71%	0.65
Treatment duration (days)					
≤28	10	1.53 [1.19, 1.87]	<0.001	55%	
>28	5	0.93 [0.37, 1.49]	0.001	70%	0.07
Postprandial measurements					
MTT	12	1.38 [1.00, 1.75]	<0.001	71%	
OGTT	3	1.14 [0.72, 1.56]	<0.001	0%	0.42

DPP4, dipeptidyl peptidase 4; GIP, glucose-dependent insulinotropic polypeptide; SMD, standard mean difference; CI, confidence interval; HbA1c, glycosylated hemoglobin; BMI, body mass index; OADs, oral antidiabetic drugs; OGTT, oral glucose tolerance test; AUC, area under the curve; MTT, meal tolerance test.

### Publication bias

**Figure 3:**
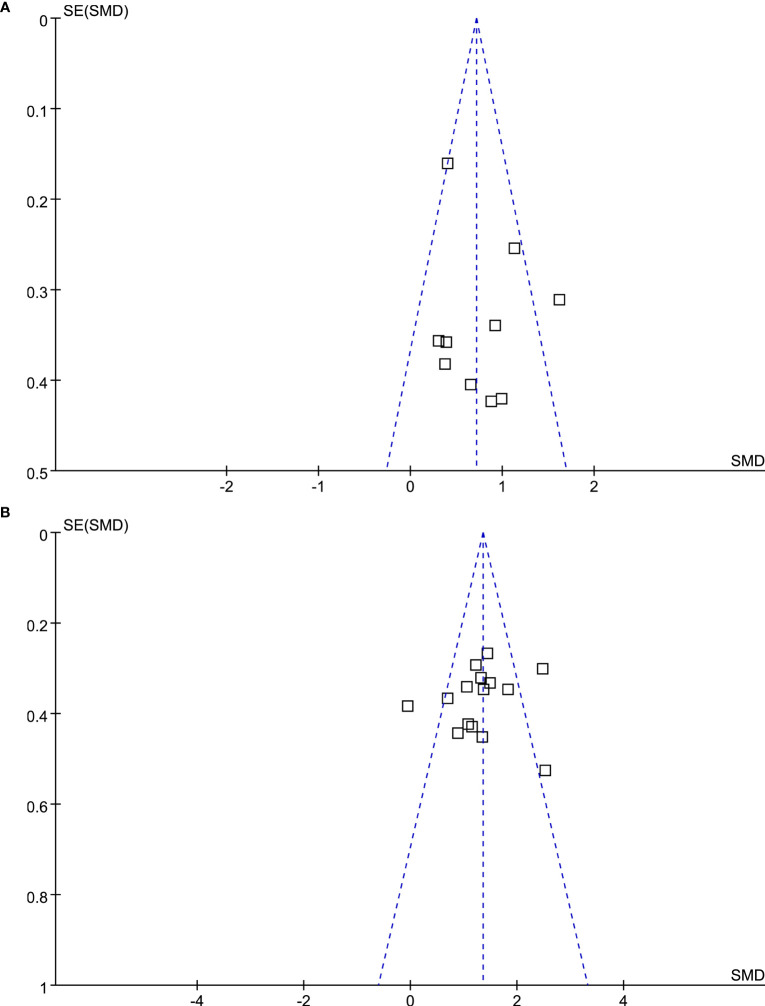
Funnel plots for the meta-analysis evaluating the influence of DPP4 inhibitors on circulating GIP in T2DM patients. **(A)** Funnel plots for the meta-analysis of the influence of DPP4 inhibitors on fasting GIP; and **(B)** funnel plots for the meta-analysis of the influence of DPP4 inhibitors on postprandial AUC_GIP_.

The funnel plots underlying the meta-analyses were symmetrical, which reflected a low risk of publication bias (**Figures 3A, B**). Egger’s regression tests also showed consistent results (*P*=0.248 and 0.515, respectively).

## Discussion

The results of the meta-analysis showed that DPP4 inhibitors significantly increased the levels of fasting and postprandial GIP compared to placebo/no treatment in T2DM patients. The robustness of the findings was further validated by consistent results of influence analysis by excluding one dataset at a time, similar results of separate sensitivity analyses limited to studies using a radioimmunoassay only, and the subgroup results according to multiple predefined study characteristics, such as study design, age, BMI, and baseline HbA1c, background antidiabetic treatments, follow-up duration, and method for the determination of the postprandial GIP level. Collectively, these findings confirmed that DPP4 inhibitors do increase active GIP concentrations in patients with T2DM.

This may be the first systematic review and meta-analysis aiming to evaluate the possible influence of DPP4 inhibitors on GIP concentrations in T2DM patients. The methodological strengths of this meta-analysis included an extensive literature search and a broad full-text review of 648 articles to identify potential eligible papers, comprehensive evaluation of the influence of DPP4 inhibitors on GIP incorporating both the fasting and postprandial GIP outcomes, and performance of multiple sensitivity and subgroup analyses to indicate the stability of the findings. Moreover, early studies showing a possible effect of DPP4 inhibitors were mostly pharmacokinetic/pharmacodynamic studies in healthy volunteers or T2DM patients treated for one day or with a single dose (32, 33), which do not accurately simulate the real-world management of patients with T2DM. All of the above considerations motivated us to perform a systematic review and meta-analysis to comprehensively determine the influence of DPP4 inhibitors on the GIP levels in T2DM patients.

As mentioned previously, it is well known that DPP4 inhibitors are OADs that regulate incretin hormones. For many healthcare professionals, appreciation of the beneficial effects of DPP4 inhibitors on incretin hormones has mainly focused on GLP-1, and the potential relevance of their influence on GIP concentrations in T2DM patients has been largely ignored. However, recent clinical trials with tirzepatide, a dual GIP and GLP-1 receptor agonist, have shown that this drug has superior efficacy in terms of the anti-hyperglycemic effects and weight loss compared to the selective GLP-1 receptor agonist semaglutide (34), thus highlighting the benefit of harnessing the actions of both GIP and GLP-1 in T2DM patients (35). Although it has been clearly shown that DPP4 inhibition increases concentrations of the intact version of both incretins in patients with T2DM (36), the relative contribution of each for the antidiabetic efficacy of DPP4 inhibitors has been uncertain, especially given the impaired insulinotropic action of GIP in this population. Studies utilizing the GLP-1 receptor antagonist, exendin 9-39 have demonstrated that not all of the glucose-lowering actions can be attributed to GLP-1 (37, 38), suggesting that the actions of GIP may be important. More recently, the importance of GIP has been confirmed, with a GIP receptor antagonist being used to demonstrate that the action of endogenous GIP accounts for around 37% of the improvement in β-cell function seen during DPP4 inhibition by sitagliptin (39).

Clinically, therefore, the influence of DPP4 inhibitors on GIP concentrations is likely to contribute to the beneficial effects of DPP4 inhibitors in patients with T2DM. For example, previous studies have shown that endogenous GIP has a greater potentiating effect on glucose-stimulated insulin secretion than endogenous GLP-1 in healthy individuals (40), and plays an important role in postprandial insulin secretion in T2DM patients (41). As mentioned above, it has now been demonstrated that endogenous GIP does contribute to beneficial effect of DPP4 inhibitors on β-cell function in patients with T2DM (39). In addition, GIP also has been shown to regulate energy disposal and storage by acting on metabolically sensitive organs, such as the adipose tissue (42). Accordingly, DPP4 inhibitors have also been demonstrated to be useful for the treatment of metabolic conditions with disordered energy homeostasis (43, 44), although their influence on body weight may not be clinically relevant.

DPP4 inhibition is associated with a low risk of hypoglycemia, which has largely been attributed to the glucose-dependency of the islet effects of GLP-1 *(i.e*. that insulin secretion is only enhanced and glucagon secretion suppressed at normal and elevated blood glucose concentration) (45). However, GIP also glucose-dependently regulates glucagon and insulin secretion in humans. Thus, like GLP-1, GIP only potentiates insulin release when glucose levels are raised (46), but its actions on glucagon differ from those of GLP-1. Accordingly, GIP does not affect glucagon concentrations during hyperglycemia, and actually enhances them under fasting or hypoglycemic conditions (46). Therefore, it may be speculated that DPP4 inhibitor-induced increases in active GIP may help to enhance glucagon levels if the blood glucose levels begin to fall into the hypoglycemic range, thereby contributing to explain the low risk of hypoglycemia associated with DPP4 inhibition. Additional studies are warranted in the future to further characterize which of the beneficial effects of DPP4 inhibitors for T2DM patients might be related to the enhanced GIP concentrations obtained following treatments.

As mentioned previously, subgroup analyses according to the study characteristics all consistently showed that compared to placebo/no treatment, DPP4 inhibitors significantly increased the levels of fasting and postprandial GIP in T2DM patients. Moreover, the increase of fasting GIP following DPP4 inhibitor administration may be apparent in T2DM patients with concurrent OAD use compared to those who were drug-naive or without concurrent OAD use. The studies with concurrent OAD use all included metformin, which has been suggested to synergistically increase the GLP-1 levels with DPP4 inhibitor administration. However, there is limited evidence on whether coadministration of DPP-4i and metformin further increases GIP concentrations compared to either drug alone (28). In the present study, for the outcome of postprandial GIP, subgroup analysis did not reveal any greater effect of DPP4 inhibitors on GIP levels in patients with concurrent OAD therapy, in whom metformin was mostly used, compared to in those who were drug-naive or without concurrent OAD use. Although the underlying mechanisms for the subgroup results have not been fully elucidated, these findings may provide an additional rationale for the combined use of DPP4 inhibitors with other OADs such as metformin in T2DM patients, which might further increase the fasting GIP level.

Our study also has some limitations that must be addressed. First, the number of studies included is limited, and the sample sizes of the included studies are generally small. Large-scale RCTs are preferable to validate the effects of DPP4 inhibitors on GIP in T2DM patients. In addition, moderate heterogeneity exists for the meta-analyses of the fasting and postprandial GIP. Although not supported by the subgroup analyses, the differences in patient and study characteristics may be important sources of heterogeneity, such as study design, age, BMI, and baseline HbA1c of the participants, background antidiabetic treatments, and follow-up duration; moreover, some other uncontrolled factors may affect the GIP level and subsequently lead to heterogeneity, such as dietary factors (47). Although the methods for measuring GIP varied among the included studies, the majority employed the same well-characterized radioimmunoassay (antibody 98171) for intact GIP (30), and sensitivity analysis limited to these studies showed consistent results with the main meta-analysis, including other methods for measuring GIP. To the best of our knowledge, no consensus has been reached regarding the gold standard for the measurement of intact GIP. Finally, the follow-up durations were relatively short among the included studies (6–168 days), which prevents from extrapolating the results to a longer period of one or more years. Longer-term influence of DPP4 inhibitors on GIP should be determined in future studies.

## Conclusions

As a summary, this meta-analysis demonstrated the effectiveness of DPP4 inhibitors for increasing the fasting and postprandial intact/active GIP levels in T2DM patients. These results further validate the hypothesis that augmentation of GIP concentration is among the multiple mechanisms of pharmacological efficacy of DPP4 inhibitors in T2DM.

## Data availability statement

The original contributions presented in the study are included in the article/supplementary material. Further inquiries can be directed to the corresponding author.

## Author contributions

SC, RZ and MY conceived, designed, or planned the study. SC, RZ and JC collected or assembled the data. SC and MY performed or supervised analyses. SC, RZ, RC, CD, YZ, SR and MY interpreted the results. SC wrote the initial draft. YZ obtained funding. All authors contributed to the article and approved the submitted version.
